# Development of genetic manipulation tools for *Pseudomonas oleovorans*

**DOI:** 10.3389/fmicb.2025.1691967

**Published:** 2025-10-30

**Authors:** Hongjiao Ke, Zhichao Zhang, Yan Liu, Quan Luo, Xuefeng Lu

**Affiliations:** ^1^College of Life Science and Technology, Harbin Normal University, Harbin, China; ^2^Key Laboratory of Photoelectric Conversion and Utilization of Solar Energy, Qingdao Institute of Bioenergy and Bioprocess Technology, Chinese Academy of Sciences, Qingdao, China; ^3^Shandong Energy Institute, Qingdao, China; ^4^Laboratory for Marine Biology and Biotechnology, Qingdao Marine Science and Technology Center, Qingdao, China

**Keywords:** *Pseudomonas*, genetic manipulation, antibiotic susceptibility, electroporation, neutral site

## Abstract

Due to the robust capabilities in hydrocarbon/herbicide degradation, biopolymer/compatible solute synthesis, steroid bioconversion, and zinc salt solubilization, *Pseudomonas oleovorans* has shown considerable potential for industrial, agricultural, and environmental applications. However, the poor availability of genetic tools for this bacterium hinders genetic, biochemical, metabolic, and engineering studies. In the present study, a genetic manipulation system that is based on electroporation was established for *P. oleovorans* strain T9AD. Antibiotic susceptibility profiling demonstrated that aminoglycoside-type antibiotics, such as kanamycin and gentamycin, are suitable selective markers. Optimization of electroporation parameters, including processing temperature for competent cell preparation, DNA concentration, DNA-cell pre-incubation, and post-pulse recovery, yielded stable electroporation efficiencies at levels of 10^4^ CFU/μg DNA. Among five candidate genomic neutral sites, two were experimentally verified and exhibited favorable suitability for gene integration. Integration of reporter genes at these sites did not affect cell growth, salt tolerance, and compatible solute anabolism. Using these neutral sites or the broad-host-range plasmid pBBR1MCS-5, regulated gene expression via the genome- or plasmid-based strategies was successfully achieved. All together, these tools, in combination with established conjugation methods, set up a robust technological platform to facilitate fundamental and application research in *P. oleovorans*.

## 1 Introduction

*Pseudomonas* spp., comprising one of the largest genera of Gram-negative bacteria, thrive in a broad spectrum of environments from soils and waters to extreme niches (Lalucat et al., 2022; Saati-Santamaría et al., 2022). Given their metabolic diversity, these bacteria exhibit remarkable potential in industrial, agricultural, and environmental applications (Craig et al., 2021; Mehmood et al., 2023). As a member with rarely reported pathogenic associations, *Pseudomonas oleovorans* displays robust capabilities in alkane degradation and biopolymer biosynthesis, such as poly (3-hydroxyalkanotes), thereby attracting considerable biotechnological interest (Huisman et al., 1991; Fiedler et al., 2002; Nisar et al., 2025). Subsequent studies have unveiled additional functions of this species, including degradation of halogenated herbicides (e.g., acetochlor; Xu et al., 2006; Chen et al., 2024), bioconversion of steroids (e.g., hydrocortisone to prednisolone; Abd El-Hady and Abd El-Rehim, 2004), solubilization of insoluble zinc salts (Rehman et al., 2021), and compatible solute synthesis (e.g., glucosylglycerol [GG]). These discoveries further expand the biotechnological relevance of *P. oleovorans* to herbicide bioremediation, biofertilizer development, pharmaceutical synthesis, and moisturizer production.

A convenient and stable genetic manipulation system in *P. oleovorans* is essential for in-depth investigations into genetic, biochemical, and regulatory mechanisms of the aforementioned functions, as well as for advancing genetic engineering studies. However, reports on specialized methods and genetic tools for *P. oleovorans* remain extremely limited. Most of these studies rely on approaches (e.g., conjugation) developed for well-characterized species such as *P. putida* (Klinke et al., 2000; Nandy et al., 2021; Guo et al., 2022). In the present study, an electroporation-based genetic manipulation system for *P. oleovorans* was established by using a marine-derived strain, T9AD (Wang et al., 2021).

## 2 Materials and methods

### 2.1 Bacterial strains and cultivation conditions

The bacterial strains used in the present study are listed in Table 1. *P. oleovorans* T9AD was grown in LB medium (for growth experiment) or M9 minimal medium (containing 0.5% wt/vol L-lactic acid as the sole carbon source, for GG production) at 30 °C. *Escherichia coli* was grown in LB medium at 37 °C. Cell growth was monitored by measuring the optical density at a wavelength of 600 nm (OD_600_). Solid media were prepared by adding 1.5% (wt/vol) agar. Kanamycin (Km) of 50 μg/mL and/or gentamycin (Gm) of 10 μg/mL were added when required.

**Table 1:** Bacterial strains and plasmids used in the present study.

Name	Characteristic or description	Source or reference
Strain		
*E. coli* DH5α	Wild-type strain, for gene cloning	Our strain collection
*P. oleovorans* T9AD	Wild-type strain, from Marine Culture Collection of China	MCCC 1A04326
KH115	*P. oleovorans* T9AD mutant, NS1::Km^r^, Km^r^	This study
KH116	*P. oleovorans* T9AD mutant, NS2::Km^r^, Km^r^	This study
KH121	*P. oleovorans* T9AD mutant, NS1::Gm^r^, Gm^r^	This study
KH122	*P. oleovorans* T9AD mutant, NS2::Gm^r^, Gm^r^	This study
KH123	*P. oleovorans* T9AD mutant, NS1::Km^r^-P_trc_-*gfp*, Km^r^	This study
KH126	*P. oleovorans* T9AD mutant, NS2::Km^r^-P_trc_-*gfp*, Km^r^	This study
KH130	*P. oleovorans* T9AD harboring pBBR1MCS-5::Km^r^-P_trc_-*lacZ*, Gm^r^Km^r^	This study
KH132	*P. oleovorans* T9AD harboring pBBR1MCS-5::Km^r^, Gm^r^Km^r^	This study
Plasmid		
pKH34	Containing the NS1::Km^r^ fragment, for constructing KH115	This study
pKH35	Containing the NS2::Km^r^ fragment, for constructing KH116	This study
pKH53	Containing the NS2::Gm^r^ fragment, for constructing KH122	This study
pKH54	Containing the NS1::Gm^r^ fragment, for constructing KH121	This study
pQL250	Containing *gfp*	Our strain collection
pQL164	Providing Gm^r^ cassette	Our strain collection
pKH55	Providing the NS1::Km^r^-P_trc_ platform	Our strain collection
pKH56	Providing the NS2::Km^r^-P_trc_ platform	Our strain collection
pKH57	Containing the NS1::Km^r^-P_trc_-*gfp* fragment, for constructing KH123	This study
pKH58	Containing the NS2::Km^r^-P_trc_-*gfp* fragment, for constructing KH126	This study
pKH63	Providing the Km^r^-P_trc_-*lacZ* fragment	Our strain collection
pBBR1MCS-5	Broad-host-range shuttle plasmid	Kovach et al. (1995)
pKH69	pBBR1MCS-5::Km^r^-P_trc_-*lacZ*, for constructing KH130	This study
pKH72	pBBR1MCS-5::Km^r^, for constructing KH132	This study

In the test of antibiotic sensitivity, *P. oleovorans* T9AD was inoculated into 96-well plates containing LB medium supplemented with different concentrations (0, 1, 2, 5, 10, 20, 50, and 100 μg/mL) of Km, ampicillin (Amp), chloramphenicol (Cm), spectinomycin (Spe), apramycin (Apr), Gm, neomycin (Neo), and streptomycin (Stp). A negative control was included with no cell inoculation. After 20 h cultivation on a horizontal shaker (MB100-2A, Hangzhou Aosheng, China) at 500 rpm, cell growth was determined by measuring the OD₆₀₀ on a microplate reader (SpectraMax M3, Molecular Devices, United States).

### 2.2 Sequence analysis

The *P. oleovorans* T9AD genome under the GenBank accession number LR130779.2 was used for sequence analysis. The manual examination of open reading frames (ORFs) was conducted using the ARTEMIS program (Rutherford et al., 2000).

### 2.3 Competent cells of *Pseudomonas oleovorans* and electroporation

The optimized procedure for competent cell preparation and electroporation was as follows: *P. oleovorans* strain T9AD was grown in 100 mL of LB medium in 250 mL flasks. Overnight cultures were harvested by centrifugation at room temperature (RT), washed twice with 0.3 M sucrose solution, and resuspended in the same solution. Electroporation was performed with the following procedure: a proper amount of plasmid DNA was added into 100 μL of competent cells to a final concentration of 0.02 μg/mL. An incubation step was not necessary. The mixtures were immediately transferred into electrocuvettes, which have a gap of 2 mm, and electropulse treated by an electroporator (Bio-Rad Micropulser, United States) with the following parameters, 12.5 kV/cm, 25 μF, 200 Ω, and a pulse duration of 5 ms. Immediately after pulses, cells were supplemented with 900 μL of SOC medium (Russell and Sambrook, 2001) and incubated at 30 °C for 1 h recovery. Then cells were plated onto LB agar plates containing antibiotics for selection. Generation of Gm-resistant (Gm^r^) and/or Km-resistant (Km^r^) transformants was examined after 20 h of cultivation. During the optimization of the electroporation method, different processing temperatures for competent cell preparation, such as 4 °C and RT, DNA concentrations (0–5 μg/mL), durations for DNA-cell incubation (0–2 h), and times for cell recovery (0–24 h) were tested.

### 2.4 DNA manipulation

The plasmids and primers used in the present study were listed in Table 1 and Supplementary Table S1, respectively. To construct mutant KH115, the upstream (0.90 kb) and downstream (0.98 kb) flanking regions of the neutral site 1 (NS1) were amplified from the total DNA of *P. oleovorans* T9AD using primer pairs NS1-up-F/NS1-up-R(Km) and NS1-dn-F(Km)/NS1-dn-R, respectively. A 0.93 kb Km^r^ cassette was amplified from pCE-Zero (Vazyme Biotech, China) using primers Km-F(NS1) and Km-R(NS1). The three fragments were assembled and cloned into pUC19 using the ClonExpress® Ultra One-Step Cloning Kit (Vazyme Biotech, China), resulting in plasmid pKH34. After sequence confirmation by sequencing, pKH34 was electrotransformed into *P. oleovorans* T9AD. After 20 h of cultivation for homologous double crossover, Km^r^ transformants were obtained on LB agar plates containing Km, and the genotypes of the transformants were confirmed by PCR. The other recombinant strains of *P. oleovorans*, including KH116 (using primers NS2-up-F/NS2-up-R(Km) and NS2-dn-F(Km)/NS2-dn-R for flanking regions), KH121 (using primers NS1-up-F/NS1-up-R(Gm) and NS1-dn-F(Gm)/NS1-dn-R for flanking regions), and KH122 (using primers NS2-up-F/NS2-up-R(Gm) and NS2-dn-F(Gm)/NS2-dn-R for flanking regions), were constructed following a similar procedure. The 1.19 kb Gm^r^ cassette was obtained from pQL164 using primers Gm-F(NS1)/Gm-R(NS1) or Gm-F(NS2)/Gm-R(NS2). For constructing KH123 and KH126, the ORF of the *gfp* gene (coding for green fluorescent protein, 0.72 kb) was amplified from pQL250 using primer pair gfp-F/gfp-R(NS1) or gfp-F/gfp-R(NS2) and cloned into pKH55 and pKH56 at the sites downstream of the *trc* promoter (P_trc_), generating plasmids pKH57 and pKH58 for electroporation. For constructing KH130, the Km^r^-P_trc_-*lacZ* cassette, amplified from pKH63 using primers KPZ-F and KPZ-R, was cloned into pBBR1MCS-5, generating pKH69 for electroporation.

### 2.5 Phenotype analyses

To determine GG production, *P. oleovorans* cells grown in M9 minimal medium were harvested by centrifugation and inoculated into 100 mL of the same medium supplemented with 3% (wt/vol) NaCl at an initial OD_600_ of 0.8. After 6 h of cultivation, GG was extracted from *P. oleovorans* cells for determination. GG extraction and quantification were performed as previously described (Qiao et al., 2018; Qiao et al., 2019).

To analyze GFP fluorescence, strains KH123 and KH126 were grown in LB medium and an isopropyl-D-1-thiogalactopyranoside (IPTG) concentration ranging from 0 to 0.25 mM was applied to induce *gfp* expression. After 6 or 20 h of induction, cells were harvested and resuspended in M9 minimal medium with an OD₆₀₀ of ~1.0. 200 μL of cell suspensions was transferred to 96-well microplates, and fluorescence intensity was measured using a microplate reader (SpectraMax M3, Molecular Devices, United States) with an excitation wavelength of 488 nm and an emission wavelength of 525 nm. Visualization of GFP fluorescence was accomplished using a fluorescence microscope (Axio Imager 2, Zeiss, Germany).

To analyze β-galactosidase (LacZ) activity, strains KH130 and KH132 were grown in LB medium and induced by adding IPTG (final concentrations of 0–0.25 mM). After 6 or 20 h of induction, β-galactosidase activity was determined following a reported protocol (Miller, 1972; Jaishankar and Srivastava, 2020; Fitzgerald et al., 2024).

## 3 Results and discussion

### 3.1 Antibiotic sensitivity profiling of *Pseudomonas oleovorans* T9AD

A clear profiling of antibiotic susceptibility forms the foundation for developing genetic manipulation tools in a given microbe, as it guides rational selection of selective markers and tool vectors (Aparicio et al., 2015). Due to the generally low outer membrane permeability and the presence of multiple efflux pumps and modification enzymes (e.g., aminoglycoside-modifying enzymes and 16S rRNA methylase) in the cell, *Pseudomonas* species generally exhibit intrinsic resistance to many antibiotics (Burns et al., 1989; Ramos et al., 2002; Daniels and Ramos, 2009; Fernández et al., 2012; Aghazadeh et al., 2013). Additionally, *Pseudomonas* can also acquire antibiotic resistance through chromosomal mutations and horizontal gene transfer (Botelho et al., 2019). For example, while *P. aeruginosa* is generally sensitive to carbapenems, some clinic isolates of this species acquire carbapenemase-encoding genes and exhibit resistance to these antibiotics (Botelho et al., 2015; Oliver et al., 2015). Therefore, for a given strain, the antibiotic resistance profile can be species- or strain- specific and requires clear determination on a case-by-case basis.

**Figure 1:**
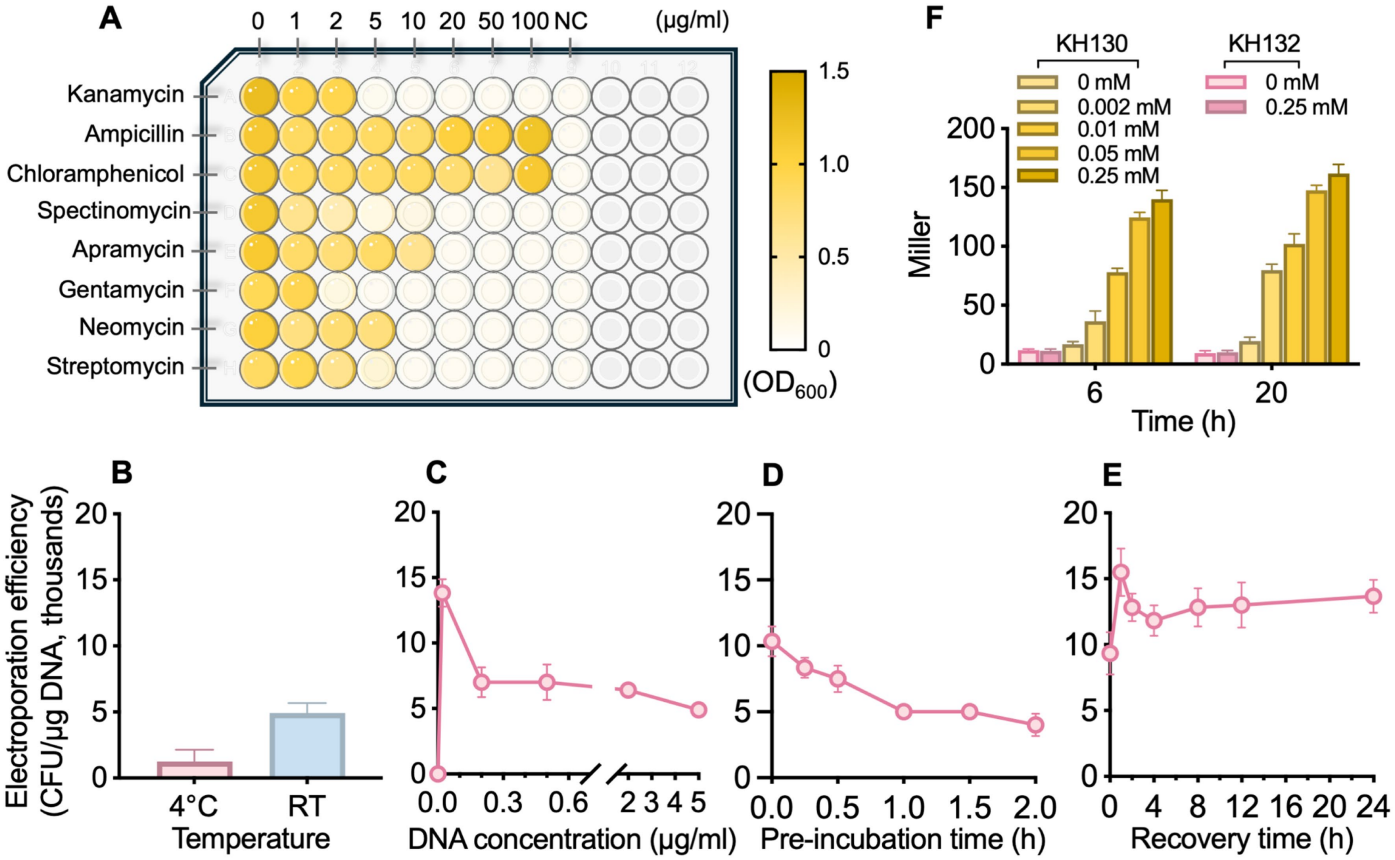
Determination of the antibiotic susceptibility profile of *P. oleovorans* T9AD **(A)**, optimization of its electroporation efficiency **(B–E)**, and assessment of plasmid-based gene expression in *P. oleovorans* T9AD **(F)**. In **(A)**, *P. oleovorans* T9AD was grown in liquid LB medium supplemented with different antibiotics (as indicated) at an initial optimal density at 600 nm (OD₆₀₀) of 0.05. After 20 h of cultivation, cell growth was monitored by measuring OD_600_. Uninoculated LB medium was used as the negative control (NC). In **(B–E)**, the effects of processing temperature for cell preparation and electropulse (**B**, 4 °C and RT), DNA concentration (**C**, 0–5 μg/mL), pre-incubation time for DNA-cell mixture (**D**, 0–2 h), and post-pulse cell recovery (**E**, 0–24 h) on the electroporation efficiency were analyzed. Each parameter was altered independently, whereas the others remained constant. In **(F)**, the β-galactosidase activity of mutants KH130 (expressing *lacZ* in pBBR1MCS-5) and KH132 (blank control) was examined. Cells were grown in LB medium, and IPTG of 0–0.25 mM was supplemented to induce *lacZ* expression. The data are presented as means from three independent replicates with standard deviations.

To determine the antibiotic susceptibility profile of *P. oleovorans* T9AD, eight commonly used antibiotics, namely Km, Amp, Cm, Spe, Apr, Gm, Neo, and Stp, at final concentrations of up to 100 μg/mL, were employed to apply selective pressure (Figure 1A; Supplementary Table S2). In liquid culture, Km and Gm exhibited the most potent inhibitory effects on cell growth. A concentration of 5 μg/mL completely arrested the growth of *P. oleovorans* T9AD, indicating a minimal inhibitory concentration (MIC) of ≤ 5 μg/mL. By contrast, Stp, Apr, Neo and Spe, showed moderate inhibition. Complete suppression of cell growth was observed at antibiotic concentrations of 20 μg/mL (for Apr, Neo, and Stp) or 50 μg/mL (for Spe). For Amp and Cm, no inhibitory effect was detected under the tested conditions. A similar inhibitory pattern was also observed in solid culture (Supplementary Figure S1). These results agreed well with the established understanding that *Pseudomonas* species are generally susceptible to aminoglycoside-type antibiotics while exhibiting resistance to β-lactam- and amphenicol-type antibiotics (Kadurugamuwa and Beveridge, 1997; Fernández et al., 2012; Hujer et al., 2023). For example, *P. aeruginosa*, *P. putida*, and *P. fluorescens* are sensitive to Gm, Km, Stp, and tobramycin. These antibiotics were frequently used in the genetic studies of *Pseudomonas* (Shivaji et al., 1989; Ghiglione et al., 1999; Wu et al., 2015; Calero et al., 2018). A key factor underlying the efficacy of these antibiotics is their strong ability to penetrate bacterial cell membranes (Lang et al., 2023). On the other hand, many *Pseudomonas* bacteria (e.g., *P. cepacia*, *P. loganensis, P. aeruginosa*, and *P. putida*) also exhibit natural resistance to Cm, carbapenems, and penicillins (Burns et al., 1989; Fernández et al., 2012; Horna et al., 2018; Karaman et al., 2025). The wide presence of RND family multidrug efflux pumps (such as MexAB-OprM and MexXY-OprM) and hyperexpression of modifying enzymes (such as AmpC β-lactamases) in the cells contribute considerably to their intrinsic antibiotic resistance by reducing intracellular drug accumulation and inactivating β-lactam compounds, respectively (Barceló et al., 2022; Lorusso et al., 2022).

### 3.2 Electroporation optimization of *Pseudomonas oleovorans* T9AD

To introduce foreign DNA into *Pseudomonas* cells, conjugation and electroporation are the most commonly used methods. The former enables the transfer of large DNA fragments but is generally more time-consuming and less efficient for small fragments compared with electroporation (Harris et al., 2016; Bai et al., 2024). To the best of our knowledge, the few genetic investigations in *P. oleovorans* have all relied on conjugation. By employing suicide vectors (e.g., pUT- and pEGM-series) and shuttle vectors (e.g., pVLT- and pHERD-series), genetic constructs have been delivered into *P. oleovorans* cells, enabling successful gene inactivation (e.g., *phaD*), chromosomal disruption (e.g., CRISPR array), expression of reporter genes (e.g., *lacZ*), and gene complementation (e.g., *phaD* and *phaF*; Table 2; Prieto et al., 1999; Klinke et al., 2000; Nandy et al., 2021; Guo et al., 2022). In comparison with conjugation, electroporation offers greater flexibility with respect to the types and lengths of foreign DNA. For example, based on this strategy, a rapid all-in-one plasmid-based CRISPR/Cas9 system was established for genome editing in *P. putida*. This allowed one edit in less than 1.5 days. Although being fast and simple for gene transfer, electroporation generally suffers from low efficiency in *Pseudomonas* species at initial attempts. Reducing nonessential genetic components in genomes and optimizing the electroporation procedure can substantially improve transformation efficiency (Choi et al., 2006; Fan et al., 2024; Wen et al., 2024). Here, the feasibility of electroporation in *P. oleovorans* T9AD was examined using the broad-host-range plasmid pBBR1MCS-5 (Kovach et al., 1995).

**Table 2:** Experimental details of genetic investigations in *P. oleovorans*.

Plasmid	Vector type and property	DNA transfer	Purpose	References
pUT-Tc	pUT-based suicide vector, containing mini-Tn*5* and *oriT* (RP4)	Conjugation	Random mutagenesis	de Lorenzo et al. (1990); Prieto et al. (1999)
pPG132	pUT-based, *Pc_I_*::*lacZ*	Conjugation	Mini-Tn*5*-insertion-based expression of *lacZ*	
pPF61	pUT-based, *lacI*^q^-*Ptrc*::*phaF*	Conjugation	Gene complementation of *phaF*	
pUT-*phaD*::*tet*	Using pUT backbond, *phaD*Ω(8 bp::*tet*)	Conjugation	For *phaD* inactivation	de Lorenzo et al. (1993); Klinke et al. (2000)
pHAD5	pVLT-based shuttle vector, RSF1010, *phaD*	Conjugation	Gene complementation of *phaD*	
pminiTn7Gm-lacItac-*ofp*	^–a^	Conjugation	Labeling *P. oleovorans* ICTN13 with orange fluorescent protein (*Ofp*)	Nandy et al. (2021)
pMSL13	pEMG-based suicide vector, *oriT* (RP4), *traJ*, *oriV* (R6K)	Conjugation	Site-specific insertion of the *mNeonGreen* gene	Qiu et al. (2008); Guo et al. (2022)
pMSL15	pEMG-based	Conjugation	For deletion of the CRISPR array	
pHERD30T	pUCP30T derivative, shuttle vector, *araC-P_BAD_*, *ori* (pBR322), *ori* (pRO1600), *oriT*	Conjugation	Expression of target crRNA	

a Not available.

Our initial attempt of electrotransforming *P. oleovorans* T9AD yielded an efficiency of only 66.7 CFU/μg DNA, a level that was too low for efficient genetic manipulation. Therefore, a systematically optimization of electroporation efficiency was conducted. Optimization began with the following settings: (i) preparing electrocompetent cells at 4 °C, (ii) using 1.5 μg/mL plasmid DNA, (iii) no pre-incubation of DNA with cells, and (iv) 4 h-recovery after pulses. Each parameter was modified individually while the others remained constant. In many cases, low temperatures were used for competent cell preparation and electroporation pulses (Diver et al., 1990; Qin et al., 2022). However, some studies have also reported improved electroporation efficiencies at RT for *Pseudomonas* species (Choi et al., 2006; Tu et al., 2016). Here, a similar result was observed in *P. oleovorans* T9AD (Figure 1B). When competent cells were prepared and pulsed at RT, the electroporation efficiency increased 3.8-fold compared with the level of 4 °C. The value raised from 1.3 × 10^3^ to 4.9 × 10^3^ CFU/μg DNA. Therefore the RT condition was applied in the following experiments. In the assessment of the relationship between electroporation efficiency and DNA concentration, the highest efficiency (1.4 × 10^4^ CFU/μg DNA) was observed at a concentration of 0.02 μg/mL, followed by a sharp decline at 0.2 μg/mL (Figure 1C). At higher concentrations (≥ 0.2 μg/mL), the value continued to decrease gradually. Thus, low DNA concentrations favor efficient electroporation in *P. oleovorans*. Similar findings have been reported in *Rhodobacter sphaeroides*, *Nocardia nova*, and *Bacillus subtilis* (Luo et al., 2013; Zhang et al., 2015; Lee et al., 2024).

The effects of DNA-cell pre-incubation and cell recovery were further examined. With an increase of incubation time, the number of positive transformants exhibited a decreasing tendency (Figure 1D). Compared with the non-incubated control (0 h), a 2 h pre-incubation reduced the efficiency by half. Therefore, a pre-incubation of competent cells with DNA prior to electroporation is unnecessary. Regarding the process after electropulses, a recovery of pulse-treated cells in antibiotic-free medium seems helpful to increase efficiency (Figure 1E). In all recovery attempts with different times (0–24 h), increased numbers of transformants were observed in comparison with the control (0 h). The highest level (1.6 × 10^4^ CFU/μg DNA) was achieved after 1 h recovery. From five randomly selected transformants, plasmids were extracted and examined by restriction digestion. They all exhibited identical pattern to the parent plasmid in the electroporation analysis (Supplementary Figure S2). Taken together, these optimizations yielded stable electroporation efficiencies of 10^4^ CFU/μg DNA in *P. oleovorans*, sufficient for genetic manipulations such as gene inactivation or expression.

### 3.3 Controllable gene expression on a plasmid platform

For genetic and metabolic engineering studies in *Pseudomonas*, stable and controllable gene expression platforms are required due to the needs of gene complementation, heterologous protein expression, pathway construction, metabolic flux regulation, and other applications. These are usually accomplished by plasmid-based systems and genomic integration.

Plasmid-based systems usually offer advantages in expression flexibility (e.g., temporal control and intensity regulation) and ease of manipulation (Rangwala et al., 1991; Blatny et al., 1997). Several types of shuttle vectors have been validated for use in *Pseudomonas* (Table 3). The pBBR1MCS-series plasmids, known for their broad-host-range, compact size, moderate copy number, and multiple cloning sites, have been validated for stable heterologous gene expression in many Gram-negative bacteria including *Pseudomonas* species (Elzer et al., 1995; Battisti and Minnick, 1999; Takahashi et al., 2016). They could be ideal candidates for gene or pathway expression in *P. oleovorans*. During electroporation optimization, the stable and autonomous replication of pBBR1MCS-5, a Gm^r^ version of pBBR1MCS, in *P. oleovorans* has been demonstrated. Thus, this plasmid was further employed to drive inducible expression of the *lacZ* reporter gene via the commonly used *E. coli trc* promoter (P_trc_) in strain T9AD. Upon plasmid transformation, a dose-dependent induction of *lacZ* expression was observed in the recombinant strain KH130 (Figure 1F). It displayed β-galactosidase activity in the presence of IPTG, with enzyme activity increasing progressively along with elevated IPTG concentrations. In contrast, the control strain KH132, which harbored a blank plasmid, exhibited only basal activity regardless of IPTG concentration. These results validated the utility of pBBR1MCS-based vectors and the *trc* promoter for controllable expression of non-native genes in *P. oleovorans*. Considering that *P. oleovorans* exhibits sensitivity to Km, Apr, Spe, Stp, and tetracycline (Figure 1A; Prieto et al., 1999), other pBBR1MCS variants harboring appropriate resistant cassettes (e.g., pBBR1MCS-2 and pBBR1MCS-3; Kovach et al., 1995) could be also applied. In addition to the broad-host-range capability, pBBR1MCS plasmids also demonstrate good compatibility with other broad-host-range plasmids from the IncP, IncW, and IncQ groups. This property is particular useful for synthetic biology investigations, as it enables flexible co-expression of multiple genes or pathways using plasmids from different incompatibility groups (such as those listed in Table 3; Antoine and Locht, 1992; Kovach et al., 1994; Li et al., 2020).

**Table 3:** Selected examples of shuttle vectors used in *Pseudomonas*.

Plasmid	Vector type	Replicon for *Pseudomonas* (Inc. group)^b^	Used in	Purpose	References
pUCP18, 19	^–a^	pRO1600 (−)	*P. aeruginosa*	Construction of *E. coli*-*Pseudomonas* shuttle vector	Schweizer (1991)
pKG228	pUCP-based	pRO1600 (−)	*P. putida*	Gene complementation of *rpoN*	West et al. (1994); Jones et al. (2024)
pHERD20T, 26 T, 28 T, 30 T	pUCP-based	pRO1600 (−)	*P. aeruginosa* *P. fluorescens* *P. putida*	For inducible gene expression via *araC*-*P_BAD_* system	Qiu et al. (2008)
pBBR1MCS	pBBR1-based	pBBR1 (−)	*P. fluorescens* *P. putida*	Developing broad-range shuttle vectors	Kovach et al. (1994); Kovach et al. (1995)
pEBP41	pBBR1-based	pBBR1 (−)	*P. putida*	Developing novel shuttle vectors for gene expression in different bacterial hosts	Antoine and Locht (1992); Troeschel et al. (2012)
pHAD5	pVLT-based	RSF1010 (IncQ)	*P. putida* *P. oleovorans*	Gene complementation of *phaD*	de Lorenzo et al. (1993); Klinke et al. (2000)
pMMB67-*nadD2*	pMMB66EH-based	RSF1010 (IncQ)	*P. aeruginosa*	Overexpression of *nadD2*	Fürste et al. (1986); Jin et al. (2019)
pMON5757	pMON7051-based	RSF1010 (IncQ)	*P. fluorescens* *P. putida* *P. testosterone* *P. syringae*	Developing shuttle vectors for efficient gene expression in different Gram-negative bacteria	Barry (1988); Rangwala et al. (1991)
pME6031::*pmeR*^+^	pME6031-based	pVS1 (−)	*P. syringae*	Gene complementation of *pmeR*	Heeb et al. (2000); Lee et al. (2025)

a Not available.

b Inc. Group, incompatibility group.

### 3.4 Development of genomic neutral platforms in *Pseudomonas oleovorans*

**Figure 2:**
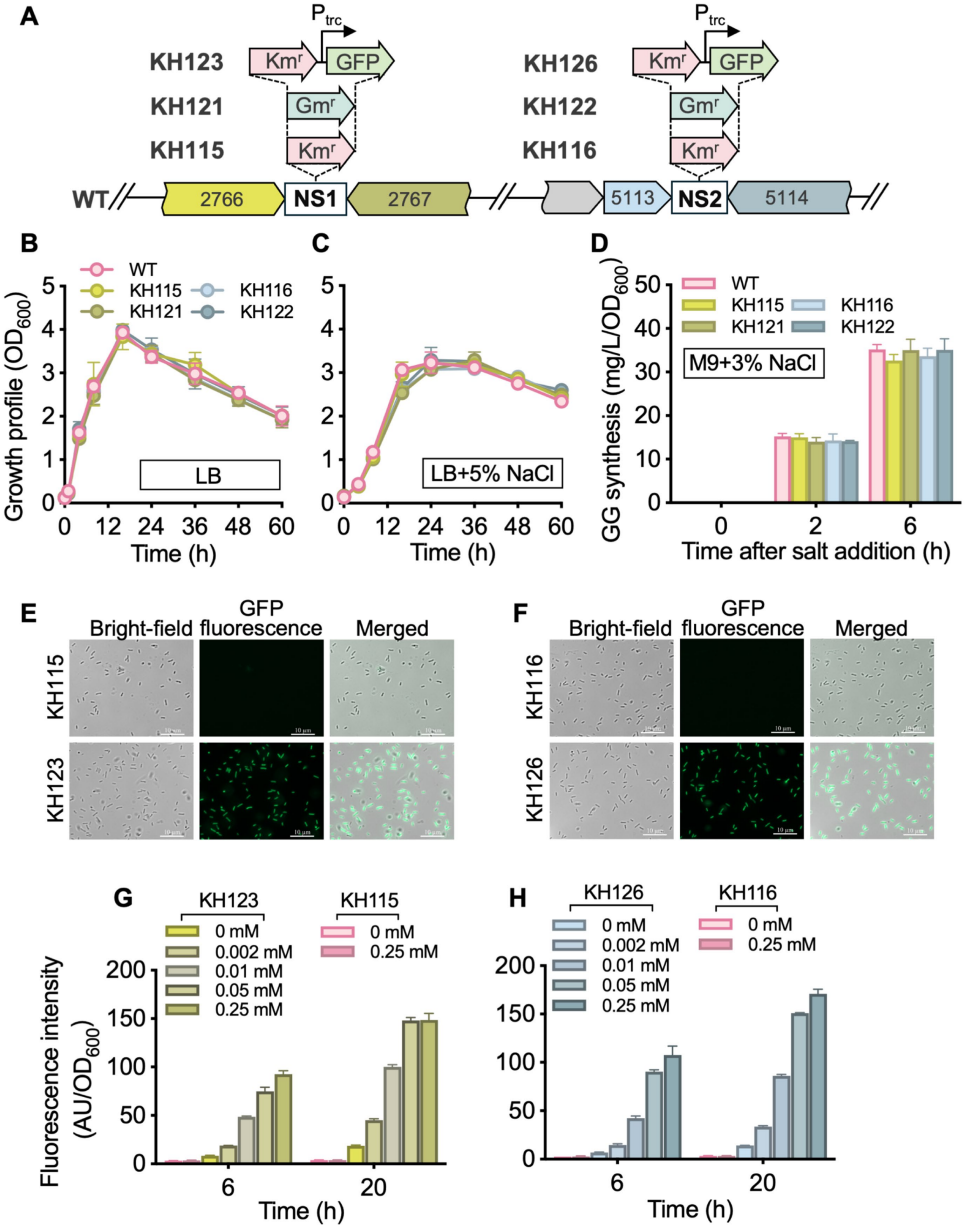
Development of genomic NSs in *P. oleovorans* T9AD. **(A)** Illustrates the genomic loci of NS1 and NS2 in the wild-type (WT) strain and the insertion of foreign genes in these sites. KH115, KH116, KH121, KH122, KH123, and KH126 are the resulting insertion mutants. The prefix “POT9AD_” of locus tags POT9AD_2766, POT9AD_2,767, POT9AD_5113, and POT9AD_5,114 is omitted. Growth profiles of the WT and mutant strains in the absence and presence of 5% salinity are demonstrated in **(B,C)**, respectively. *Pseudomonas* cells were grown in LB medium with or without 5% (wt/vol) NaCl addition. **(D)** Demonstrates the GG-synthesizing ability of the WT and mutant strains of *P. oleovorans* T9AD. Cells were grown in M9 minimal medium and 3% (wt/vol) NaCl was added to induce GG production. In **(E–H)**, GFP fluorescence of mutants KH123 and KH126 were examined. Cells were grown in LB medium, and IPTG of 0–0.25 mM was supplemented to induce *gfp* expression. The data of **(B–D)** and **(G,H)** are presented as means from three independent replicates with standard deviations. Km^r^, Km-resistant; Gm^r^, Gm-resistant; P_trc_, the *trc* promoter.

Genomic NSs are useful tools for bacterial genetic and metabolic engineering studies. Insertion of genes in these chromosomal loci does not cause detectable phenotypic changes of cells. Since no NSs have been identified in *P. oleovorans* to date, the genome of strain T9AD was analyzed for this purpose and four criteria were considered: (i) chromosomal regions lacking predicted ORFs; (ii) region of 250–300 bp in length to minimize the presence of other possible functional elements, (iii) flanking genes with opposite transcriptional directions facing each other, and (iv) the absence of similar sequences to the target NS and its flanking genes elsewhere in the genome. Five candidate sites of 256–292 bp in length were identified (Supplementary Figure S3), and two of them with the longest flanking genes, NS1 (POT9AD_2766–2,767) and NS2 (POT9AD_5113–5,114), were selected to investigate their neutrality. A Km^r^ gene and a Gm^r^ gene were independently introduced into these sites via homologous recombination (Figure 2A). The resulting mutants KH115, KH116, KH121, and KH122 exhibited the expected antibiotic-resistances. Regardless of whether the medium contained 5% NaCl or not, the mutant strains showed growths comparable to that of the wild-type strain (Figures 2B,C). Thus, integration of foreign genes into NS1 and NS2 did not affect the basic growth of *P. oleovorans* T9AD as well as its salt-tolerance. During genome analysis, it was found that *P. oleovorans* T9AD harbors a putative glucosylglycerol (GG) synthetic pathway encoded by the GG-phosphate phosphatase/synthase gene *ggpPS* (Cheng et al., 2024), suggesting this strain might synthesize GG as compatible solute for salt acclimation. The GG-synthesizing capability of T9AD was examined (Figure 2D). Under the condition of 3% NaCl, salt-induced GG production was observed in the wild-type and mutant strains. The mutant strains accumulated GG levels comparable to the wild-type. Thus gene insertion in NS1 and NS2 did not influence GG anabolism of *P. oleovorans*.

Compared to plasmid-based gene expression, the genome-integrated system eliminates the need to consider plasmid loss, plasmid incompatibility, and host range limitations. It also allows cultivation without the addition of antibiotics (Friehs, 2004; Bassalo et al., 2016; Li et al., 2020; Kazi et al., 2022). To investigate the feasibility of NS1 and NS2, the green fluorescent protein reporter gene (*gfp*) was integrated into these sites under the control of the *trc* promoter (Figure 2A). Under fluorescence microscopy, the recombinant strains KH123 and KH126 showed strong fluorescence, indicating successful *gfp* expression (Figures 2E,F). The fluorescence intensity correlated positively with increasing IPTG concentrations (0 to 0.25 mM) and induction durations (6 to 20 h; Figures 2G,H). Under the same conditions, no GFP signal was detected in the control strains (KH115 and KH116; Figures 2E–H). These results demonstrated that stable and dose-dependent expression of non-native genes or pathways could be achieved via the *trc* regulatory system within the NS1 and NS2 platforms. Previously, chromosome-based gene integration in *Pseudomonas*, such as *P. fluorescens and P. putida*, has mainly been achieved employing the *mini*Tn*7*-transposition system (Koch et al., 2001; Lambertsen et al., 2004). This requires a miniTn*7* vector and relies on the presence of the *att*Tn*7* site, a neutral intergenic region located immediately downstream of the glucosamine-6-phosphate synthetase gene (*glmS*). In *P. oleovorans*, an *att*Tn*7*-like site is detected in the genome, and the newly developed NS1 and NS2 sites offer alternative options alongside *att*Tn*7*, making them suitable for multi-module expression.

## 4 Conclusion

In summary, this study established an electroporation-based genetic system for *P. oleovorans*, a species with biotechnological potential. Aminoglycoside antibiotics, especially Km and Gm, showed potent inhibitory effects on cell growth, providing a rational basis for selective marker design. By optimizing the temperature for cell preparation and electropulse, DNA concentration, and post-pulse cell recovery, the electroporation efficiency was improved to stably achieve the level of ~10^4^ CFU/μg DNA. Employing the broad-host-range vector pBBR1MCS-5, plasmid-based inducible expression of foreign genes could be achieved. Additionally, two genomic NSs (NS1 and NS2) were developed and validated for gene integration without major phenotypic influence. Collectively, these tools, along with established conjugation method, set up a robust technological platform to facilitate further fundamental and application studies in *P. oleovorans*.

## Data Availability

The data presented in this work are available upon request from the authors.
