# Supplementary material for: Development of genetic manipulation tools for Pseudomonas oleovorans
Source: Front Microbiol. 2025 Oct 30;16:1691967. doi: 10.3389/fmicb.2025.1691967 (PMC12612858; doi:10.3389/fmicb.2025.1691967)

(Supplementary Material)

**Development of genetic manipulation tools for *Pseudomonas oleovorans***

Hongjiao Ke^1,2^, Zhichao Zhang^2,3^, Yan Liu^1*^, Quan Luo^2,3*^, Xuefeng Lu^2,3,4*^

^1^College of Life Science and Technology, Harbin Normal University, Harbin, China

^2^Key Laboratory of Photoelectric Conversion and Utilization of Solar Energy, Qingdao Institute of Bioenergy and Bioprocess Technology, Chinese Academy of Sciences, Qingdao, China

^3^Shandong Energy Institute, Qingdao, China

^4^Laboratory for Marine Biology and Biotechnology, Qingdao Marine Science and Technology Center, Qingdao, China

**^*^Correspondence:**

Xuefeng Lu; Email: lvxf@qibebt.ac.cn; Tel.: +86–532–80662712

Quan Luo; Email: luoquan@qibebt.ac.cn; Tel.: +86–532–80662711

Yan Liu; Email: yanliuhrb@hotmail.com; Tel.: +86–451–88060576

**Table S1** Primers used in the present study.

| Primer | Sequence (5’-3’) |
| --- | --- |
| NS1-up-F | GAGCTCGGTACCCGGGGATCGGCACCCTGCGCGAGCCGTTC |
| NS1-up-R(Km) | GCCCCAGCTGGCAATTCCGGCGGTTCGATCTGCAAATGAG |
| NS1-up-R(Gm) | GTGTCCCCTTATACACAAGGACGGTTCGATCTGCAAATGAG |
| NS1-dn-F(Km) | CTTCTTGACGAGTTCTTCTGACTGTCGGAACGAATTTTCGTC |
| NS1-dn-F(Gm) | GCCGAGATCGGCTTCCCGGCCTGTCGGAACGAATTTTCGTC |
| NS1-dn-R | CTGCAGGTCGACTCTAGAGGCGACGTTCCTTTCCCCAGAATC |
| Km-F(NS1) | CTCATTTGCAGATCGAACCGCCGGAATTGCCAGCTGGGGC |
| Km-R(NS1) | GACGAAAATTCGTTCCGACAGTCAGAAGAACTCGTCAAGAAG |
| Gm-F(NS1) | CTCATTTGCAGATCGAACCGTCCTTGTGTATAAGGGGACAC |
| Gm-R(NS1) | GACGAAAATTCGTTCCGACAGGCCGGGAAGCCGATCTCGGC |
| NS2-up-F | GAGCTCGGTACCCGGGGATCCGGCAGCAGAATCCGCCCAG |
| NS2-up-R(Km) | GCCCCAGCTGGCAATTCCGGCCACAAGCTGAATAGGGCCC |
| NS2-up-R(Gm) | GTGTCCCCTTATACACAAGGACCACAAGCTGAATAGGGCCC |
| NS2-dn-F(Km) | CTTCTTGACGAGTTCTTCTGAGTGGCAGTGGTTCCTGCACTG |
| NS2-dn-F(Gm) | GCCGAGATCGGCTTCCCGGCGTGGCAGTGGTTCCTGCACTG |
| NS2-dn-R | CCTGCAGGTCGACTCTAGAGATGCCGCCTACATTGAATGC |
| Km-F(NS2) | GGGCCCTATTCAGCTTGTGGCCGGAATTGCCAGCTGGGGC |
| Km-R(NS2) | CAGTGCAGGAACCACTGCCACTCAGAAGAACTCGTCAAGAAG |
| Gm-F(NS2) | GGGCCCTATTCAGCTTGTGGTCCTTGTGTATAAGGGGACAC |
| Gm-R(NS2) | CAGTGCAGGAACCACTGCCACGCCGGGAAGCCGATCTCGGC |
| gfp-F | CTTTAAGAAGGAGATATACCATGAGTAAAGGAGAAGAAC |
| gfp-R(NS1) | GACGAAAATTCGTTCCGACAGCTATTTGTATAGTTCATCC |
| gfp-R(NS2) | CAGTGCAGGAACCACTGCCACCTATTTGTATAGTTCATCC |
| KPZ-F | CTCATTTGCAGATCGAACCGCCGGAATTGCCAGCTGGGGC |
| KPZ-R | CGAAAATTCGTTCCGACAGGCGAAATACGGGCAGACATG |

**Table S2** Original data of cell growth (OD_600_) of *P. oleovorans* T9AD in the antibiotic susceptibility profiling experiment.

| Concentration  (μg/ml)  Antibiotic | 0 | 1 | 2 | 5 | 10 | 20 | 50 | 100 | NC^a^ |
| --- | --- | --- | --- | --- | --- | --- | --- | --- | --- |
| Kanamycin | 1.29 | 1.04 | 1.02 | 0.07 | 0.06 | 0.06 | 0.06 | 0.06 | 0.06 |
| Ampicillin | 1.16 | 0.98 | 0.99 | 0.97 | 0.95 | 1.05 | 1.09 | 1.22 | 0.05 |
| Chloramphenicol | 1.14 | 0.99 | 0.98 | 0.97 | 0.97 | 0.93 | 0.84 | 1.15 | 0.05 |
| Spectinomycin | 1.17 | 0.85 | 0.71 | 0.47 | 0.44 | 0.24 | 0.06 | 0.06 | 0.05 |
| Apramycin | 1.15 | 0.96 | 0.92 | 0.97 | 0.84 | 0.05 | 0.06 | 0.06 | 0.05 |
| Gentamycin | 1.01 | 1.02 | 0.47 | 0.05 | 0.05 | 0.05 | 0.06 | 0.05 | 0.05 |
| Neomycin | 1.09 | 0.89 | 0.93 | 0.90 | 0.27 | 0.06 | 0.06 | 0.05 | 0.05 |
| Streptomycin | 0.98 | 1.01 | 0.85 | 0.57 | 0.22 | 0.05 | 0.05 | 0.05 | 0.05 |

^a^NC, negative control with no cell inoculation.


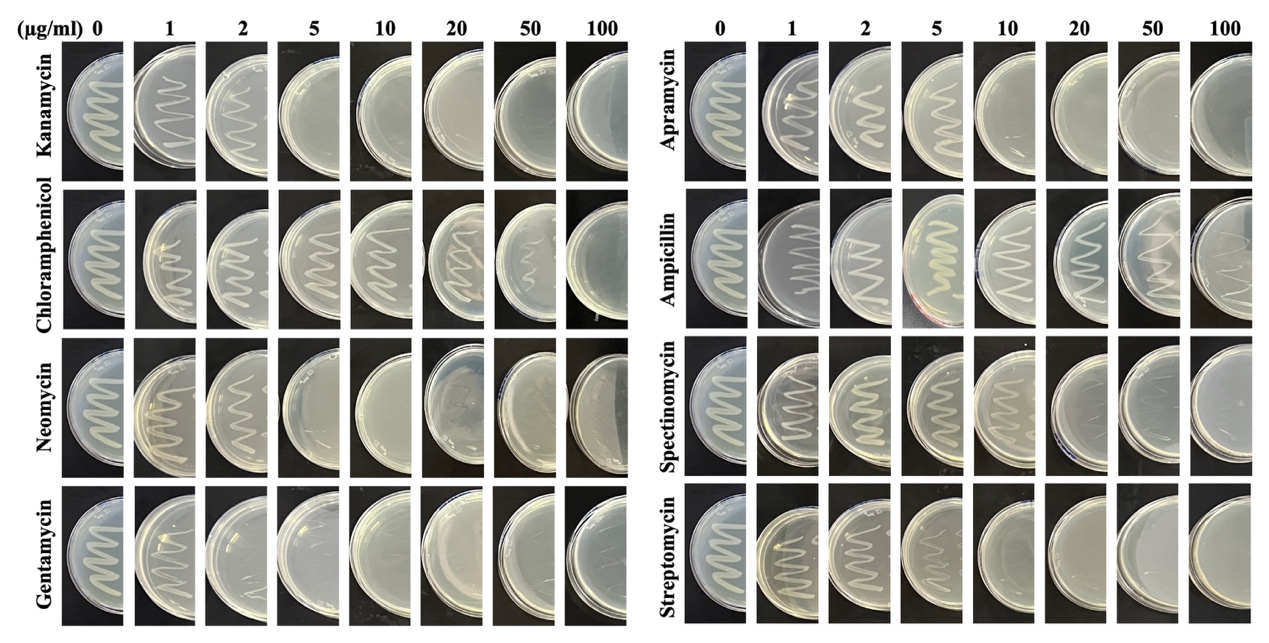


**Supplementary Figure S1.** Determination of the antibiotic susceptibility profile of *P. oleovorans* T9AD in solid culture. *P. oleovorans* T9AD cells were grown on LB agar medium supplemented with different concentrations of kanamycin, chloramphenicol, neomycin, gentamycin, apramycin, ampicillin, spectinomycin, and streptomycin as indicated. After 20 h of cultivation, cell growth was recorded.


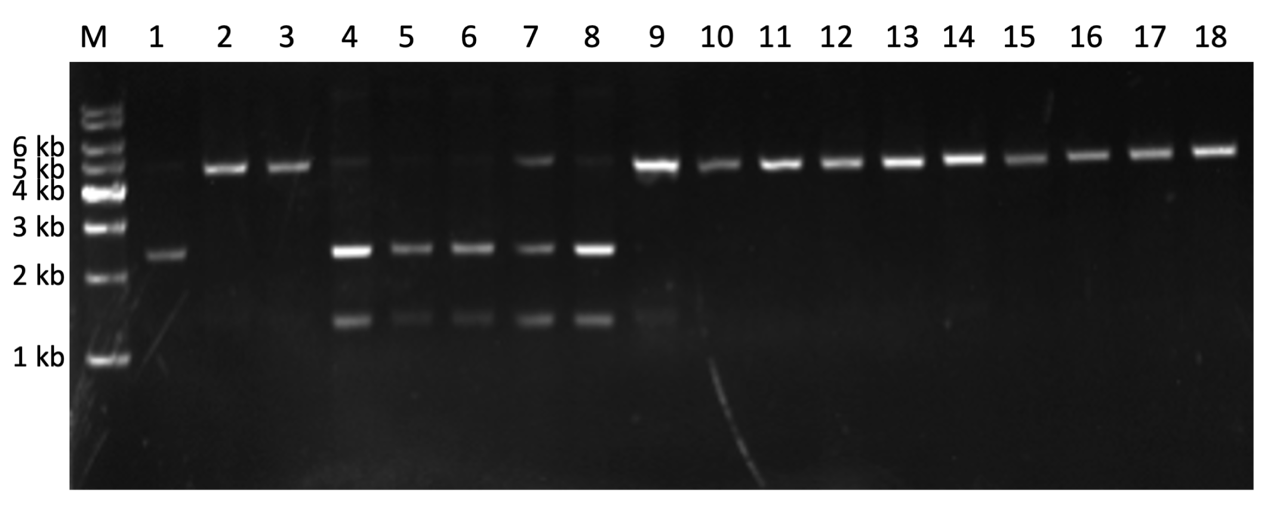


**Supplementary Figure S2.** Examination of the pBBR1MCS-5 plasmid extracted from randomly selected Gm^r^ transformants during electroporation optimization. In the restriction digestion analysis, the parent and daughter plasmids of pBBR1MCS-5 were digested using EcoRI or BamHI. Plasmids or digestion products were analyzed by agarose gel electrophoresis. M, DNA marker; line 1, parent pBBR1MCS-5; line 2, parent pBBR1MCS-5 digested by EcoRI; line 3, parent pBBR1MCS-5 digested by BamHI; line 4–8, daughter plasmids extracted from five randomly selected transformants; line 9–13, daughter plasmids digested by EcoRI; line 14–18, daughter plasmids digested by BamHI.

**Supplementary Figure S3.** Graphic illustration of the genomic loci of five candidate neutral sites (NSs) in the genome of *P. oleovorans* T9AD. The prefix “POT9AD_” of locus tags is omitted. NS of POT9AD_1328–1329, 280 bp; NS of POT9AD_2766–2767, 277 bp; NS of POT9AD_3193–3194, 292 bp; NS of POT9AD_4060–4061, 277 bp; NS of POT9AD_5113–5114, 256 bp.


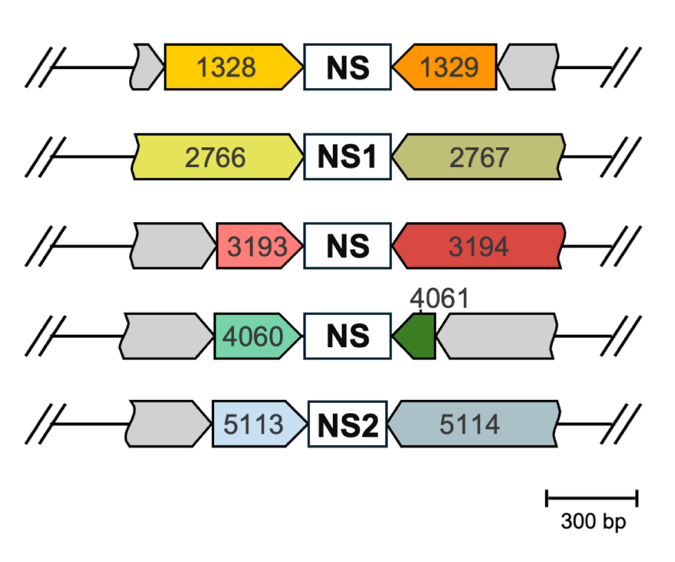

Supplement: Supplementary file 1 [file Data_Sheet_1.docx]
